# Two cases of colorectal liver metastasis with residual liver recurrence after a long recurrence-free survival period

**DOI:** 10.1186/s40792-023-01779-5

**Published:** 2023-11-21

**Authors:** Shotaro Yagi, Makoto Takahashi, Taiki Tsuji, Susumu Yanagibashi, Taku Higashihara, Hideo Ohtsuka, Tatsuya Hayashi, Kunio Takuma, Yasuhiro Morita, Ayano Nakazono, Haruka Okada, Masayuki Ohtsuka

**Affiliations:** 1 Department of Surgery, Tokyo Metropolitan Tama Medical Center, 2-8-29, Musashidai Fuchu-Shi, Tokyo, 183-8524 Japan; 2 Department of Gastroenterology, Tokyo Metropolitan Tama Medical Center, Tokyo, Japan; 3 Department of Pathology, Tokyo Metropolitan Tama Medical Center, Tokyo, Japan; 4 Department of General Surgery, Graduate School of Medicine, Chiba University, Chiba, Japan

**Keywords:** Colorectal cancer, Liver metastasis, Late recurrence, Surgery, Chemotherapy

## Abstract

**Background:**

The rate of residual liver recurrence after the resection of colorectal liver metastases is high, and most cases recur within 5 years of the initial hepatectomy. Here, we report two cases of residual liver recurrence after radical resection of colorectal liver metastases after a long recurrence-free survival period.

**Case presentation:**

Case 1 involved a 62-year-old woman treated for ascending colon cancer in April 2011 who underwent right hepatectomy for synchronous colorectal liver metastasis in April 2012. However, in September 2021, computed tomography revealed residual recurrence in the lateral segment of the liver, and a lateral segmentectomy of the liver was performed. In Case 2, a 52-year-old man treated for cecal cancer in July 2002 underwent lateral segmentectomy of the liver for metachronous colorectal liver metastasis in October 2006. Subsequently, there was no recurrence; however, computed tomography showed residual liver recurrence in the right lobe of the liver in October 2021, and an expanded posterior hepatic segmentectomy was performed.

Histopathological findings in both cases were consistent with colorectal liver metastases.

**Conclusions:**

We encountered two cases in which residual liver recurrence was observed after a long period of recurrence-free survival. Although rare, there have been a few cases of late recurrence of liver metastases after radical resection of cancer liver metastases.

## Background

Colorectal liver metastasis (CRLM) is the most common site of recurrence after radical resection of colorectal cancer. The best treatment for the first CRLM is hepatectomy, and the 5-year survival rate is reported to be 35–58% [1]. Furthermore, recurrence occurs in approximately two-thirds of cases after hepatectomy, and approximately one-third are localized in the liver. Therefore, treatment for residual liver recurrence is important [2, 3].

Residual liver recurrence after CRLM surgery often occurs within 5 years [4].

In this study, we report two cases of residual liver recurrence following CRLM resection with a long period of recurrence-free survival.

## Case presentation

### Case 1

A 62-year-old woman with no particular medical history was diagnosed with ascending colon cancer and referred to our hospital. Her blood test results were as follows; carbohydrate antigen 19–9 (CA19-9), 48.3 U/mL (< 37.0); and carcinoembryonic antigen (CEA), 11.4 ng/mL (< 5.0). She underwent laparoscopic-assisted right hemicolectomy (D3 dissection) in April 2011. Although a hepatic mass was observed preoperatively, only colectomy was performed because a definitive diagnosis of metastasis could not be made. Histopathological findings revealed that T4aN1 was a well-differentiated adenocarcinoma. She received oral uracil and tegafur plus leucovorin (UFT/LV) therapy (UFT at a dose of 370 mg/m^2^ and LV at a dose of 75 mg/body on days 1–28, every 35 days for five courses) as adjuvant chemotherapy.

On April 2012, computed tomography (CT) showed a tumor lesion with irregular margins and low density in liver segments 5 and 8. We determined that the hepatic tumor was a synchronous liver metastasis, and a right hepatectomy was performed after portal vein embolization. Four CRLM lesions were noted in the excised right liver lobe. The pathological stage was T4aN1M1a stage IVA, according to the eighth edition of the TNM classification of the American Joint Committee on Cancer (AJCC) for International Cancer Control (UICC).

She received six courses of combination therapy with capecitabine plus oxaliplatin (XELOX: capecitabine at a dose of 1,500 mg/m^2^ and oxaliplatin at a dose of 120 mg/m^2^ on day 1 of a 21-day cycle) as adjuvant chemotherapy. She has been recurrence-free for a long time and discontinued regular hospital visits in March 2019.

**Fig. 1:**
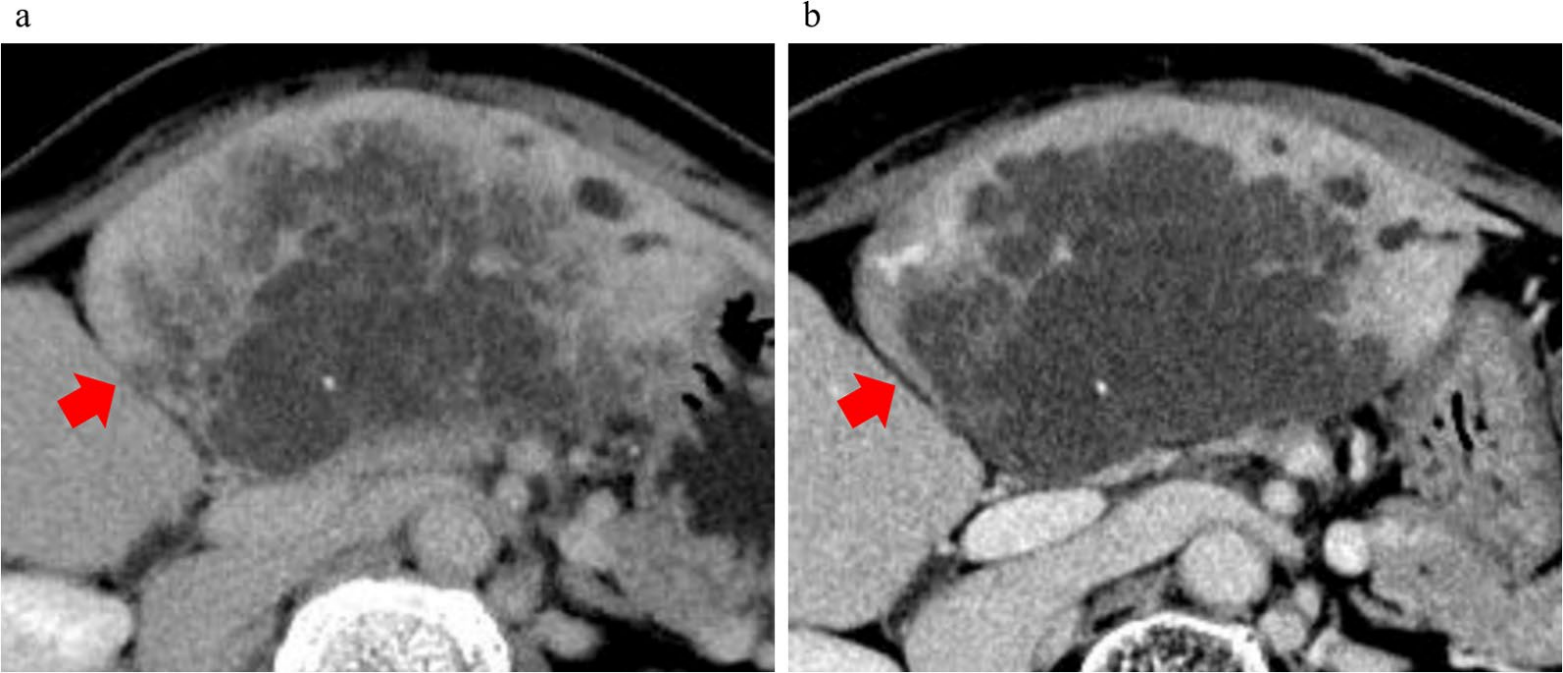
**a** Image findings of contrast-enhanced dynamic-CT before neoadjuvant chemotherapy (NAC) of Case 1. Image showed a 11 × 7.5 cm cystic lesion in the lateral segment of the liver with a faint ring-enhancement (red arrow). **b** Image findings of contrast-enhanced dynamic-CT after NAC of Case 1. The shrinkage of the tumor was not observed (red arrow). Response Evaluation Criteria in Solid Tumors (RECIST) is stable disease

In September 2021, a liver mass was incidentally noted on CT performed for a detailed examination of chronic bronchitis, and residual liver recurrence was suspected. The CA19-9 and CEA tumor marker levels had increased to 291.9 U/mL and 314.8 ng/mL, respectively. CT showed an 11 × 7.5 cm mass in the lateral segment of the liver with faint ring enhancement (Fig. 1a).

Colonoscopy did not reveal any newly developed malignant tumors. Because the tumor was large and bordered the portal umbilicus, neoadjuvant chemotherapy (NAC) was initiated. However, we could not determine whether the lesion was a late-onset CRLM recurrence or a new lesion, such as an intrahepatic cholangiocarcinoma; therefore, a liver biopsy was performed.

As a result, cytokeratin (CK) 7, CK20, and caudal-type homeobox transcription factor (CDX)2 were positive on immunohistochemical staining. Since it was similar to the primary tumor, we diagnosed it as late-onset CRLM. She was treated with triplet chemotherapy with fluorouracil/folinic acid, oxaliplatin, and irinotecan (FOLFOXIRI: oxaliplatin, irinotecan, and 5-fluorouracil at a dose of 85, 160, and 3,000 mg/m^2^, respectively) plus bevacizumab four times every 2 weeks, and combination therapy with 5‐fluorouracil, levofolinate, and irinotecan (FOLFIRI) plus bevacizumab twice and FOLFIRI once. After NAC, the liver metastasis did not shrink; however, necrosis was observed (Fig. 1b).

**Fig. 2:**
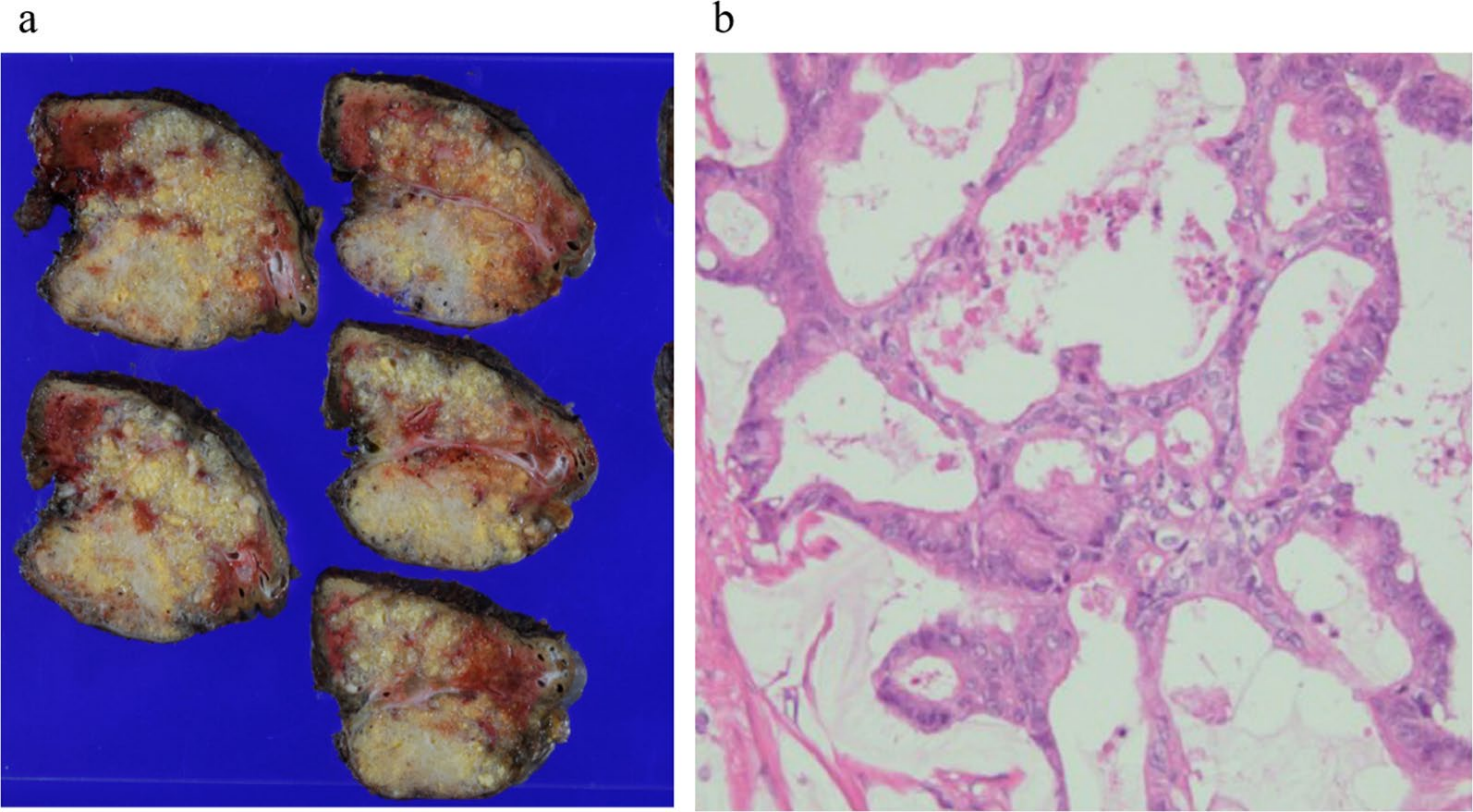
**a** Macroscopic findings of Case 1. A white mass was observed in the lateral segment of the liver. **b** Histopathological examination of Case 1 revealed extensive mucus production and partial luminal structures of atypical glandular epithelial cells

**Fig. 3:**
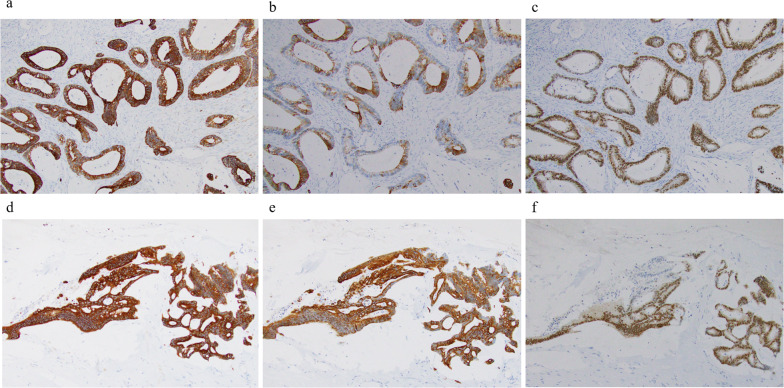
Immunohistochemical findings of Case 1. **a** Colon cancer cells were positive for cytokeratin (CK) 7. **b** Colon cancer cells were positive for CK20. **c** Colon cancer cells were positive for caudal-type homeobox transcription factor (CDX) 2. **d** Liver tumor cells were positive for CK7. **e** Liver tumor cells were positive for CK20. **f** Liver tumor cells were positive for CDX2

**Fig. 4:**
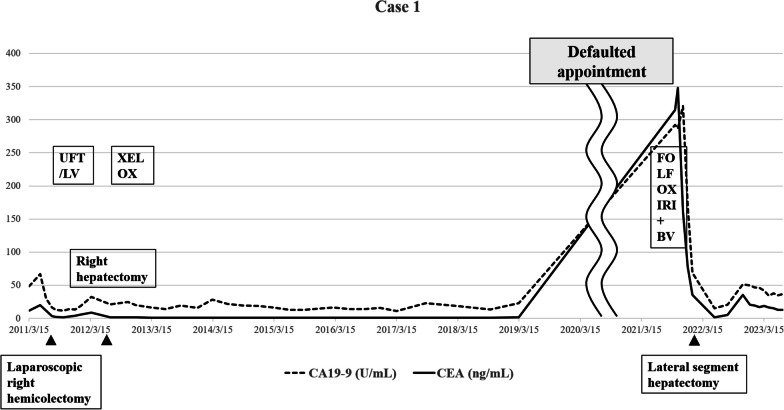
Clinical course of Case 1

She underwent lateral segmentectomy of the liver in February 2022. Although the residual liver only comprised segments 1 and 4, it had enlarged sufficiently after the previous right hepatectomy; the estimated residual liver volume was 705 cm^3^, which was sufficient for tolerating surgery. Histopathological examination revealed extensive mucus production and partial luminal structures of atypical glandular epithelial cells (Fig. 2a, b). Immunohistochemical staining showed that CK7, CK20, and CDX2 were positive in both the primary lesion of the colon and the metastatic lesion of the liver (Fig. 3a–f). This was similar to the findings for the primary tumor; therefore, she was diagnosed with metachronous CRLM. No postoperative complications were observed, and she was discharged on the eighth postoperative day. However, 6 months after the surgery, CT and revealed multiple liver metastases, and she is currently (17 months after the last surgery) undergoing chemotherapy. We have summarized the clinical course of Case 1 in Fig. 4.

### Case 2

A 52-year-old man with no relevant medical history was referred to our hospital and was diagnosed with cecal cancer. His blood test results were as follows: CA19-9, 2 U/mL, and CEA, 2.2 ng/mL.

In July 2002, he underwent ileocecal resection. The histopathological finding was a well-to-moderately differentiated adenocarcinoma, T3N0M0, stage IIA, according to the eighth edition of the TNM classification of the AJCC/UICC.

On October 2006, CT showed a low-density tumor lesion in the lateral segment of the liver. We diagnosed him with CRLM and performed a lateral segmentectomy of the liver in December 2006. Subsequently, there was no recurrence, and follow-up was completed 5 years after the first CRLM operation in December 2011.

**Fig. 5:**
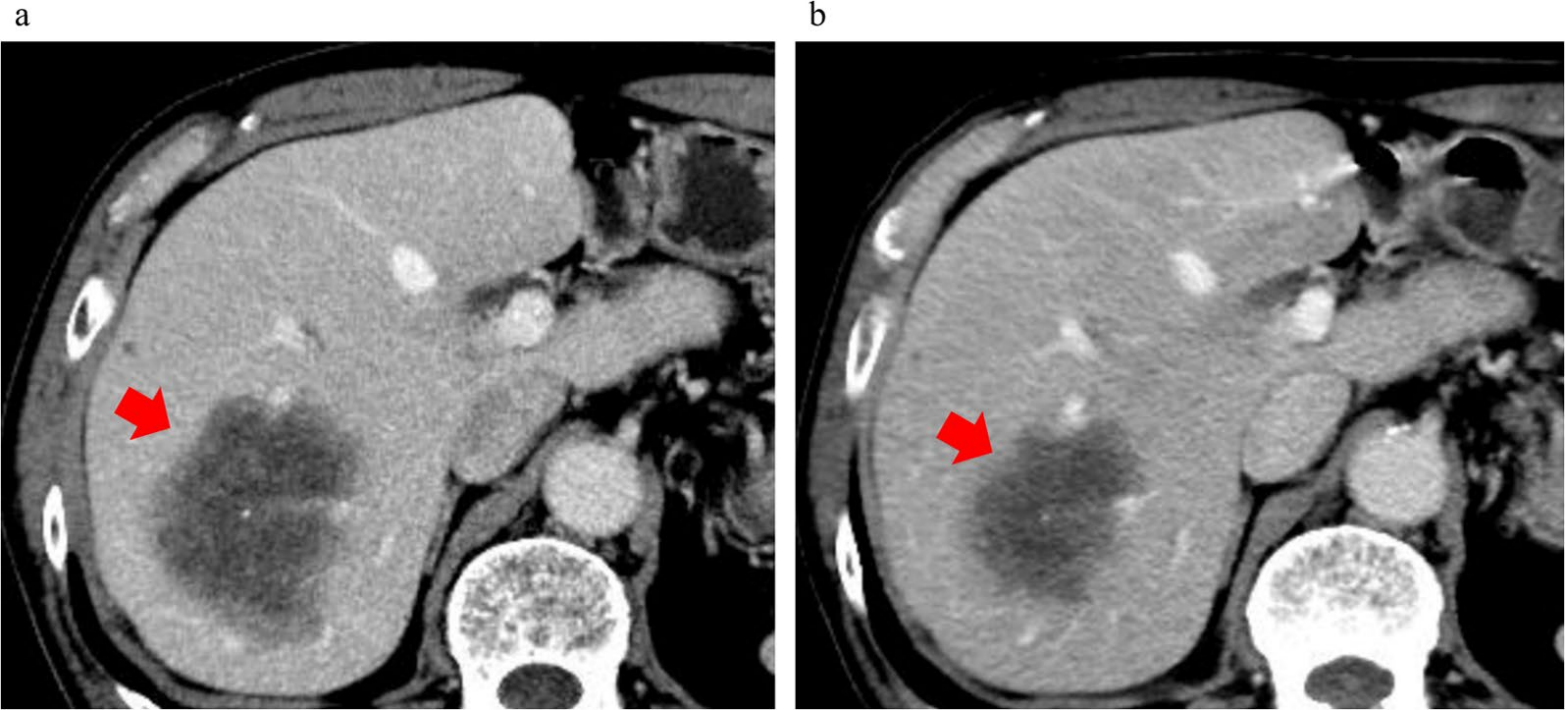
**a** Image findings of contrast-enhanced dynamic-CT of Case 2 before NAC. There was a mass of 75 mm in diameter with low density in the central part and a faint ring-enhancement effect in the S7/8 of the liver (red arrow). **b** After NAC, the tumor shrank (red arrow)

However, in November 2021, a tumor was found in S8 of the liver on ultrasonography during a medical examination, and residual liver recurrence was suspected. His blood test results were as follows: CA19-9, 15.9 U/mL; and CEA, 371.3 ng/mL. CT showed a tumor (diameter: 75 mm) with low density in the central part and a faint ring-enhancement effect in S7/8 of the liver. (Fig. 5a).

Colonoscopy did not reveal any newly developed malignant tumors.

Based on these imaging findings, he was diagnosed with metachronous CRLM. As the tumor had invaded the root of the 8 dorsal branch, we decided to perform NAC to reduce the resection area. The patient received three courses of combination therapy with capecitabine and oxaliplatin (XELOX) (capecitabine at a dose of 1,900 mg/m^2^ and oxaliplatin at a dose of 130 mg/m^2^ on day 1 of a 21-day cycle).

**Fig. 6:**
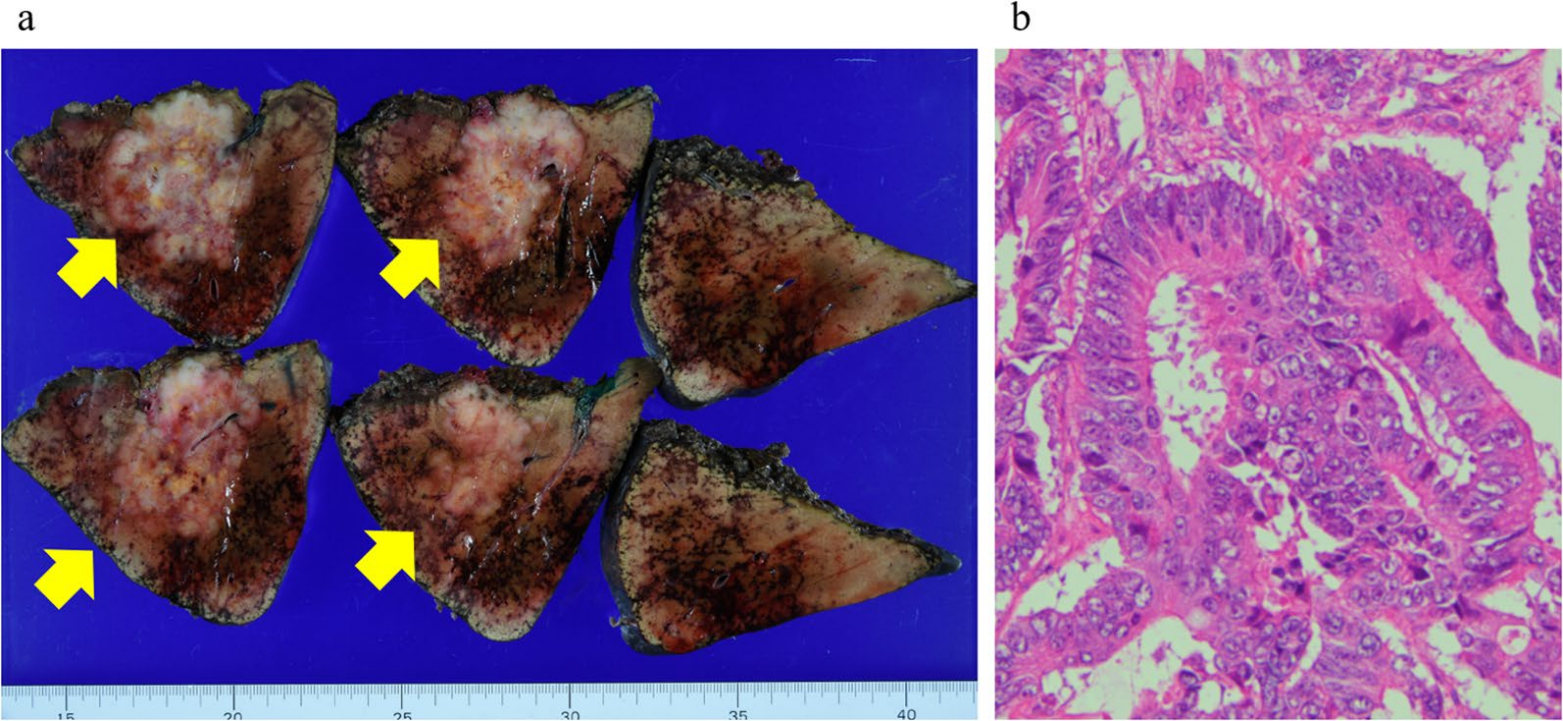
**a** Macroscopic findings of posterior segment of the liver of Case 2. A white mass was observed. **b** Histopathological examination revealed a high columnar atypical epithelium with nuclear atypia, densely forming irregular ducts, infiltrating and proliferating

**Fig. 7:**
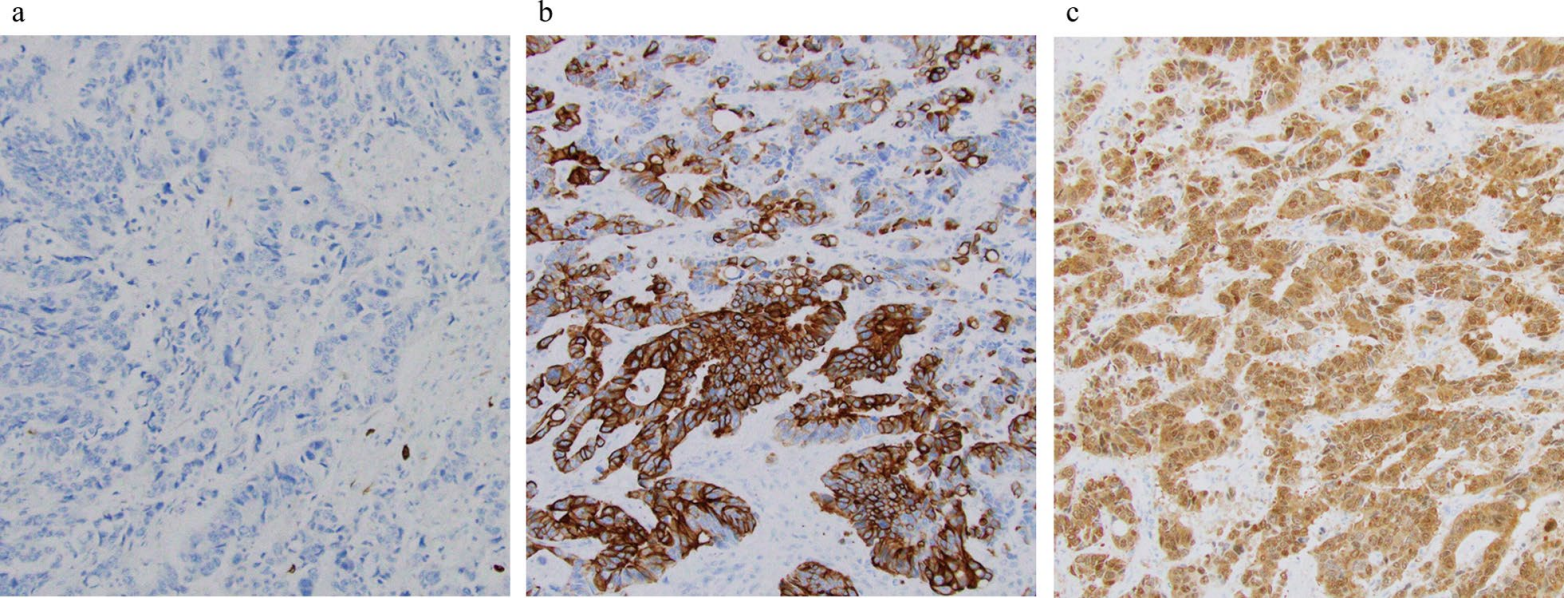
Immunohistochemical findings of Case 2. **a** Live tumor cells were negative for CK7. **b** Live tumor cells were positive for CK20. **c** Live tumor cells were positive for CDX2

After NAC, CT showed that the tumor had shrunk (Fig. 5b). Therefore, he underwent an expanded posterior segmentectomy of the liver in March 2022. Histopathological examination revealed a highly columnar atypical epithelium with nuclear atypia, densely formed irregular ducts, and infiltration and proliferation (Fig. 6a, b). Immunostaining was negative for CK7 and positive for CK20, CDX2 (Fig. 7a–c), mucin (MUC)-1, and CK19, consistent with CRLM.

**Fig. 8:**
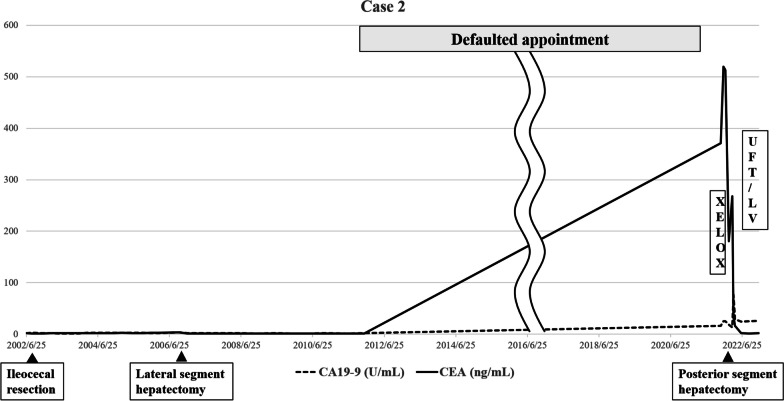
Clinical course of Case 2

No postoperative complications were observed, and he was discharged on the eighth postoperative day. After resection of the CRLM, he received UFT/LV therapy (UFT at a dose of 320 mg/m^2^ and LV at a dose of 75 mg/body on days 1–28, every 35 days for five courses) as adjuvant chemotherapy for 6 months. He has been recurrence-free for 14 months since the last surgery. We have summarized the clinical course of Case 2 in Fig. 8.

## Discussion

Because most recurrences after colorectal cancer surgery occur within 5 years, follow-up for approximately 5 years after radical surgery for colorectal cancer is recommended [4, 5]. However, there have also been reports of recurrence for more than 5 years after radical resection of colorectal cancer.

Seo et al*.* reported that 4.3% of patients with recurrent colorectal cancer had recurrence more than 5 years after the initial radical surgery. Among recurrences within 5 years, liver metastases were the most common, and among recurrences after 5 years, lung metastases were the most common [6]. Cases of lung metastases recurring more than 10 years after a curative resection for colorectal cancer have also been reported [7, 8].

Conversely, cases of liver metastases recurring more than 10 years after a radical resection for colorectal cancer have been very rarely reported [9]. In particular, only two cases (including Case 2) are known, wherein the interval between the first CRLM and the second CRLM was of more than 10 years (i.e., 15 years in both) [10].

After resection of CRLM, 50–70% of patients develop recurrent CRLM again [3]. Subsequent CRLM often occurs within 5 years of the first CRLM. Battula et al*.* performed repeat hepatectomy in 58 of the 1,027 cases with CRLM and reported that the period from the initial hepatic resection to repeat hepatectomy was 15.4 months (range, 4.3–60.1 months) [11]. Metcalfe et al*.* reported that the interval between the first and second surgeries in patients undergoing hepatectomy was reported to average 20 months, and approximately 30% of patients underwent a second surgery within 1 year [12]. However, in a Japanese study, 7% of CRLM cases relapsed 5 years after surgery [13].

We investigated cases of residual liver recurrence in patients who underwent initial resection for CRLM at our hospital for 10 years from 2006 and 2015. A total of 62 cases of residual liver recurrence were found, of which 60 cases recurred within 5 years, excluding the two cases (3.2%) in this study. The two cases recurred 9 and 15 years after resection of the primary CRLM. Table 1 summarizes cases wherein the interval between the first and second CRLM was of more than 9 years. These three cases have nothing in common other than involving relatively younger patients with primary tumors of a well differentiated histological type. Furthermore, the cases are not similar in terms of the following: (1) number and sizes of the CRLM lesions and (2) whether lymph node metastases were present or absent, whether the first CRLM was synchronous or metachronous, and whether tumor marker levels were elevated.

**Table 1:** Cases wherein the interval between the first and second CRLM was of more than 9 years

Case	Interval between first CRLM and second CRLM	Age	Sex	T	N	M	Stage	Differentiation	CA19-9	CEA	Number of first CRLM	Size of first CRLM
1	9y	62	Female	4a	1	1	IVA	Well	48.3	11.4	4	32 mm, 30 mm, 13 mm, 10 mm
2	15y	52	Male	3	0	0	IIA	Well to moderate	2	2.2	1	30 mm
Nagano et. al. [10]	15y	57	Female	3	1	1	IVA	Well to moderate	Unknown	Unknown	2	73 mm, 10 mm

Not limited to CRLM, patients in the late-recurrence group had lower preoperative CEA levels. In addition, tumors with expanding growth, well-differentiated histological types, and a lack of lymph node metastasis were more common in the late recurrence group [6]. Cho et al. stated that male predominance, the left colon or rectum as the tumor site, a grossly polypoid type, and small-sized tumors were more common in the late recurrence group [14].

Re-hepatectomy is generally a highly difficult operation because of adhesions, anatomical displacement, and changes in the liver parenchyma caused by chemotherapy. However, Wurster et al*.* and many others reported that there was no significant difference in morbidity and operative mortality rates after re-hepatectomy compared to initial hepatectomy [15].

In Case 1, the patient discontinued regular hospital visits, which delayed the discovery of the second CRLM. In Case 2, the second CRLM was found during a physical examination.

Even after surgery for CRLM, recurrence of the residual liver may occur in the late stage; therefore, follow-up should be considered even 5 years after surgery, especially in cases of younger patients with well-differentiated tumors.

## Conclusions

In this study, we encountered two cases in which residual liver recurrence was observed after a long recurrence-free survival period following radical resection of CRLM.

In patients who have undergone resection of CRLM, it is desirable to encourage them to undergo medical examinations even after completion of follow-up observation and to strive for early detection of residual liver recurrence.

## Limitation

The first operation in Case 2 was performed approximately 20 years ago, and the photographic data of the pathological specimen had already disappeared.

## Data Availability

Data sharing is applicable to this article.

## Abbreviations

CRLM: Colorectal liver metastasis
CA19-9: Carbohydrate antigen 19–9
CEA: Carcinoembryonic antigen
UFT/LV: Oral uracil and tegafur plus leucovorin
CT: Computed tomography
AJCC: American Joint Committee on Cancer
UICC: Union for International Cancer Control
XELOX: Combination therapy of capecitabine plus oxaliplatin
FOLFIRI: Combination therapy of 5‐fluorouracil, levofolinate and irinotecan
MUC: Mucin
